# The Principal Uses of the Sixteen Most Important and Fourteen Supplementary Homœopathic Medicines

**Published:** 1889-08

**Authors:** 


### THE PRINCIPAL USES OF THE SIXTEEN MOST IMPORTANT AND FOURTEEN SUPPLEMENTARY HOMŒOPATHIC MEDICINES. London : E. Gould & Son, 1889.

The purpose of this little volume is to give the "uses" of these thirty remedies for domestic practice. The volume, like most of its kind, is practically useless. Too many diseases are considered, and none are thoroughly done. If these works were devoted exclusively to the consideration of a few of the common, mild complaints, like colds, diarrhœa, headache, toothache, etc., etc., they might be of value to the laity; but when such diseases as epilepsy are included, and space wasted which could be given to the proper consideration of mild complaints, then these "domestic" works become ridiculous and useless.

